# Moral trauma, moral distress, moral injury, and moral injury disorder: definitions and assessments

**DOI:** 10.3389/fpsyg.2025.1422441

**Published:** 2025-03-05

**Authors:** Tyler J. VanderWeele, Jennifer S. Wortham, Lindsay B. Carey, Brendan W. Case, Richard G. Cowden, Charlotte Duffee, Kate Jackson-Meyer, Francis Lu, Seth A. Mattson, Robert Noah Padgett, John R. Peteet, Jonathan Rutledge, Xavier Symons, Harold G. Koenig

**Affiliations:** ^1^Human Flourishing Program, Institute for Quantitative Social Science, Harvard University, Cambridge, MA, United States; ^2^Department of Epidemiology, Harvard T.H. Chan School of Public Health, Boston, MA, United States; ^3^School of Psychology and Public Health, La Trobe University, Melbourne, VIC, Australia; ^4^Department of Psychiatry and Behavioral Sciences, University of California Davis Medical School, Sacramento, CA, United States; ^5^Baylor College of Medicine, Houston, TX, United States; ^6^Department of Psychiatry, Harvard Medical School, Boston, MA, United States; ^7^Department of Psychiatry & Behavioral Sciences, Duke University School of Medicine, Durham, NC, United States; ^8^Department of Medicine, Duke University School of Medicine, Durham, NC, United States

**Keywords:** moral trauma, moral distress, moral injury, moral injury disorder, PTSD - posttraumatic stress disorder

## Abstract

We propose new definitions for moral injury and moral distress, encompassing many prior definitions, but broadening moral injury to more general classes of victims, in addition to perpetrators and witnesses, and broadening moral distress to include settings not involving institutional constraints. We relate these notions of moral distress and moral injury to each other, and locate them on a “moral trauma spectrum” that includes considerations of both persistence and severity. Instances in which moral distress is particularly severe and persistent, and extends beyond cultural and religious norms, might be considered to constitute “moral injury disorder.” We propose a general assessment to evaluate various aspects of this proposed moral trauma spectrum, and one that can be used both within and outside of military contexts, and for perpetrators, witnesses, victims, or more generally.

## 1 Introduction

The concepts of moral injury and moral distress have received increasing attention in the scientific literature. There are now a number of, sometimes divergent, definitions and assessments concerning each of these concepts (Jinkerson, 2016; Carey and Hodgson, 2018; Koenig et al., 2019; Giannetta et al., 2020), and the scope of application of these concepts have continued to expand over time (Kälvemark et al., 2004; Fourie, 2015; Hodgson and Carey, 2017; Morley et al., 2021). In this paper we seek to (i) build on the current consensus definitions of ‘moral injury’ so as to also include moral injury that may arise from being a victim (Carey and Hodgson, 2018; Atuel et al., 2021; Litz et al., 2022), such as in cases of abuse or adverse childhood experiences, extending beyond settings of perpetrators and witnesses; (ii) relate definitions of moral injury to those of moral distress and argue that with the broader definitions of moral distress that appear in the literature, the relation between moral injury and moral distress is made clearer; and (iii) use a new expanded definition of moral injury and its relationship to moral distress to propose that these concepts may be seen on a spectrum of “moral trauma,” for which we also propose a unified approach to assessment.

## 2 The concept of moral injury

### 2.1 Prior definitions

Early descriptions of moral injury were given by Shay (1994, 2002) in documenting phenomena occurring in veterans; the idea was then further developed in the scientific literature by Litz et al. (2009). In their work, Litz and colleagues drew upon social-cognitive theories of posttraumatic stress disorder (PTSD), and conceptualized moral injury as involving an act of transgression (either perpetrated, witnessed, or experienced as an act of betrayal) that violated deeply held assumptions and beliefs about right and wrong or personal goodness. The exposure to such a transgressive act is sometimes known as a potentially morally injurious event (PMIE). Exposure to PMIEs can create dissonance between what was experienced and what was morally expected, resulting in lasting emotional, cognitive, behavioral, social, and spiritual sequelae. The core cognitive and emotional effects have generally been placed under the categories of guilt and shame. The concept has since been expanded outside of military contexts (Levinson, 2015).

Since 2009, proposed definitions of moral injury have proliferated and researchers have created several instruments to measure exposure to potentially morally injurious events (PMIEs) and subsequent moral injury symptoms. It is not our intent here to provide a systematic review of this literature but to point toward prior work synthesizing this literature and the resulting attempts at consensus definitions. We will also note potential criticism of those consensus definitions and attempt to offer refinements to address those criticisms. The present authors met regularly over the course of a year to discuss these prior consensus definitions and potential refinements.

A recent review of the measures used in military populations identified 22 distinct measures of either exposure to PMIEs, moral injury symptoms, or both (Koenig et al., 2019). Measures focusing on PMIE exposure often distinguish between perpetration-based events, witnessing an event (and not intervening), and acts of betrayal. Measures of moral injury symptoms tend to focus on the feelings of guilt, shame, loss of trust of others and institutions, loss of meaning, difficulty forgiving self and others, self-condemnation, and struggles with religious or spiritual faith. While originally developed for veterans, multiple measures have been contextualized and applied outside of military settings, including, for example, to healthcare professionals and first responders (Litam and Balkin, 2021; Mantri et al., 2020; Morris et al., 2022; Norman et al., 2023) and such phenomena can of course arise in general and civilian populations as well.

Given the diversity of definitions, Carey and Hodgson (2018), based upon the research of Shay (2002), Litz et al. (2009), Jinkerson (2016) and of Hodgson and Carey (2017), proposed a definition intended to capture numerous, sometimes divergent, aspects of moral injury. Their proposed definition was as follows:

“Moral injury is a trauma related syndrome caused by the physical, psychological, social and spiritual impact of grievous moral transgressions, or violations, of an individual’s deeply-held moral beliefs and/or ethical standards due to: (i) an individual perpetrating, failing to prevent, bearing witness to, or learning about inhumane acts which result in the pain, suffering or death of others, and which fundamentally challenges the moral integrity of an individual, organization or community, and/or (ii) the subsequent experience and feelings of utter betrayal of what is right caused by trusted individuals who hold legitimate authority.

The violation of deeply-held moral beliefs and ethical standards—irrespective of the actual context of trauma—can lead to considerable moral dissonance, which if unresolved, leads to the development of core and secondary symptoms that often occur concurrently. The core symptoms commonly identifiable are: (a) shame, (b) guilt, (c) a loss of trust in self, others, and/or transcendental/ultimate beings, and (d) spiritual/existential conflict including an ontological loss of meaning in life. These core symptomatic features, influence the development of secondary indicators such as (a) depression, (b) anxiety, (c) anger, (d) re-experiencing the moral conflict, (e) social problems (e.g., social alienation) and (f) relationship issues (e.g., collegial, spousal, family), and ultimately (g) self-harm (i.e., self-sabotage, substance abuse, suicidal ideation and death)” (Carey and Hodgson, 2018, p.2).

This consensus definition has been considered by some as the most ‘comprehensive definition’ thus far (e.g., Buhagar, 2021) as it captures a number of aspects of moral injury described in the prior literature including the possibility of moral injury arising from being a perpetrator or witness or simply learning about a transgression. The definition also distinguishes between the fundamental moral content of the injury as transgressing or violating “an individual’s deeply-held moral beliefs and/or ethical standards… and which fundamentally challenges the moral integrity of an individual, organization or community,” from the core symptoms of “(a) shame, (b) guilt, (c) a loss of trust in self, others, and/or transcendental/ ultimate beings, and (d) spiritual/existential conflict including an ontological loss of meaning in life” and distinguishes these yet further from the “secondary symptoms,” that may result from the core symptoms. Those secondary symptoms or consequences of moral injury may include “(a) depression, (b) anxiety, (c) anger, (d) re-experiencing the moral conflict, (e) social problems (e.g., social alienation) and (f) relationship issues (e.g., collegial, spousal, family), and ultimately (g) self-harm (i.e., self-sabotage, substance abuse, suicidal ideation and death).”

The definition proposed by Carey and Hodgson (2018) also allows for moral injury that may result from being a victim of a transgression, provided this is accompanied by “feelings of utter betrayal of what is right caused by trusted individuals who hold legitimate authority.” While the definition covers considerable conceptual ground, the restriction (in the case of victims) to betrayal “by trusted individuals who hold legitimate authority” may be considered as overly stringent, especially when considering moral injury outside of a military context. Arguably a victim’s experience of a grievous moral transgression could fundamentally challenge the moral integrity of an individual even when perpetrators are not “trusted individuals who hold legitimate authority.” The relative neglect of victims, outside the context of betrayal by authorities, in the moral injury so that this reads literature is also reflected in the various assessments that have been employed for empirical studies of moral injury. While the available measures that have been developed include relevant assessments for perpetrators and witnesses, they often neglect victims.

### 2.2 New definition

In what follows we would like to propose a definition, and later an assessment, that draws heavily on Carey and Hodgson (2018), but includes a broader range of potential contexts for victims (Atuel et al., 2021; Litz et al., 2022), and that provides a certain symmetry, both in definition and in assessment, for victims, perpetrators, and witnesses. We will also relate our proposed definition and assessment to a concept known as “moral distress.” We will argue that moral distress, understood in a relatively broad sense, and moral injury, may be seen as part of a spectrum, which we will refer to as “moral trauma,” concerning how severe and persistent the distress arising from a particular moral event may be. The definitions here may of course be subject to further expansion and revision as the literature around moral injury and moral distress continues to advance.

Our proposed definition for moral injury is as follows:

*Moral Injury: Persistent distress that arises from a personal experience that disrupts or threatens: (a) one’s sense of the goodness of oneself, of others, of institutions, or of what are understood to be higher powers, or (b) one’s beliefs or intuitions about right and wrong, or good and evil.*

This proposed definition shares many features with the definition proposed by Carey and Hodgson (2018) by being focused on the moral content of the experience, rather than the symptoms. The general phrasing in terms of “distress” allows for a range of potential symptoms. Both definitions require a causal link between the transgression or experience and the threat to moral integrity, and allow for an experience that threatens moral integrity to arise from perpetrating or failing to prevent a transgression, or from witnessing or learning about a transgression, or from being the victim of a transgression. Both definitions are also sufficiently broad as to allow both for recent experiences, or for experiences in the past, such as with adverse childhood experiences (ACEs), to play a causal role. The experience may be the living through of a recent event, or it may be a recalling of, or a reinterpretation of, a past event or experience.

### 2.3 Definitional distinctions

There are, however, some distinctions between the two definitions. Our proposed definition does not impose further constraints for victims of a transgression, and thus includes instances when the transgression does not arise from “trusted individuals who hold legitimate authority.” That is to say, struggles with moral injury could, for example, arise for a victim in the case of being raped by someone who is a complete stranger.

Our proposed definition also does not impose the requirement that the experiences that threaten moral integrity be “moral transgressions, or violations, of an individual’s deeply-held moral beliefs and/or ethical standards.” While, in most cases, moral injury may arise from experiences that constitute transgressions, in other cases moral injury might arise from experiences that are not transgressions but struggles, or from events involving natural phenomena which are discordant with one’s worldview or involve tragic loss. With regard to experiences that are not transgressions, a person who struggles with a moral dilemma and who believes there is no morally right way forward may begin to question his or her entire moral system and understanding of right and wrong (Fleming, 2021, 2022; Molendijk, 2018). If the latter gives rise to persistent severe distress, one might argue that it would be proper to refer to this as a moral injury (Jackson-Meyer, 2022; Tessman, 2023). Likewise, experiences of individuals struggling with addiction, or alternatively with excessive scrupulosity, so as to result in persistent distress by threatening their entire moral understanding, would qualify as instances of moral injury under our definition above, even though there might not be an interpersonal transgression. Such instances would also constitute moral injury under character-based definitions concerning threats to an individual’s character or identity (Atuel et al., 2021). However, such Aristotelian or character-based approaches are not necessary to conceive of moral injury, since all that is required, as per the definition above, is *some* notion of good and evil, or right and wrong. It is also notable that in cases of moral dilemma or struggles with addiction, it is not clear that the categorization into perpetrator, witness, or victim is applicable in all such cases. With regard to natural phenomena, the witnessing of an earthquake, for example, may deeply threaten or disrupt one’s sense of the goodness of the natural order or of God’s goodness, and if this disruption is sufficiently severe so as to give rise to persistent distress, it may again be reasonable to speak of an individual or a community experiencing a moral injury. Such non-transgressive, but potentially worldview-discrepant, events may shake assumptions about the goodness of life, God, or the world at large (Currier et al., 2015; Fleming, 2022; Koenig et al., 2018; Litz et al., 2009; Norman et al., 2024).

We believe our proposed definition thus includes all instances of moral injury covered by the definition of Carey and Hodgson (2018), but expands the conceptual coverage further in the cases of victims and to certain cases which may not involve transgressions.^1^ It bears reasonably close resemblance to the recently proposed character-based definition of Atuel et al. (2021): “moral injury results when a moral failure event leads to suffering that threatens one’s character and identity.” The precise relations between the two definitions (such as instances in which one’s understanding of the goodness of an institution has been altered and causes persistent distress) would depend on one’s understanding and definition of character and identity. However, in contrast to Carey and Hodgson (2018) and Atuel et al. (2021), our proposed definition also provides somewhat greater specificity as to the manner in which the experience of transgression threatens moral integrity. The definition specifies this as a disruption or threat concerning “one’s sense of the goodness of oneself, of others, of institutions, or of what are understood to be higher powers,” or “one’s beliefs or intuitions about right and wrong, or good and evil.” This will be important in our proposal for a moral injury assessment measure.

Another important aspect of our proposed definition for moral injury is that the distress itself be persistent. Short-lived distress may severely impact a person’s life. However, if this distress were to resolve relatively quickly, it is not clear it would be proper to refer to it as moral *injury*. The *persistence* of symptoms – continuous, intermittent or periodic – seems important in order to refer to something as an “injury.” The term “injury” also suggests a certain level of severity, and this too we believe is embedded in our proposed definition since persistent distress over matters that threaten or disrupt one’s moral understanding arguably necessarily entails a certain degree of severity. We will return to matters of severity below.

Of course, in certain cases, an individual may feel considerable distress even if it is not persistent, and such cases are also important to consider. Moreover, even if initially fleeting, if such distress is recurrent, it may over time develop into something that eventually could be described as persistent distress or moral injury (Čartolovni et al., 2021). The “experience” in our proposed definition might be a single event, but could also be understood as a series of recurrent events. Said another way, a particular instance of moral distress may itself be a precursor to moral injury. In the following section we will discuss the literature and definitions concerning moral distress and relate this to our proposed definition of moral injury.

## 3 Moral distress

While moral distress is a relatively broad concept, it has received its greatest attention in the nursing literature, and in settings where the distress arises from various institutional constraints. Jameton (1984) first described moral distress in the nursing literature as follows: “Moral distress arises when one knows the right thing to do, but institutional constraints make it nearly impossible to pursue the right course of action” (Jameton, 1984). Constraints which are beyond the control of the individual give rise to distress if the individual is unable to act on what he or she perceives to be the morally correct thing to do. Jameton’s definition suggests that distress arises from two components: (1) a moral judgment that is made, followed by (2) an inability to act on that moral judgment due to institutional constraints.

Under Jameton’s definition, moral distress differs from “moral dilemma,” “moral uncertainty,” and “moral conflict,” which are scenarios in which one is not able to make a moral judgment due to the ambiguity of multiple competing options (Jameton, 1993). Put another way, moral dilemmas exist when either no answer, or more than one answer, seems morally defensible, leading to inevitable ambiguity (Kvalnes, 2019). Making a moral judgment, which is a required component of moral distress in Jameton’s definition, cannot occur in cases of moral dilemmas, uncertainties, or conflicts because of this ambiguity. Jameton differentiated dilemmas from distress because he wanted to shift the conversation regarding ethical problems that nurses were facing away from the individual and toward the external, often institutional, constraints that led to the distressing event. Using the language of dilemmas, according to Jameton, had an undue “softening” effect, redirecting potential criticism away from institutions and co-workers who had constrained the individual from acting on what they thought was right. Using the term “distress” would allow nurses to, “say outright, ‘I think what happened here was morally reprehensible, awful, harmful, and the actions of professionals thinking more of their own pocketbooks and achievements than of the patient’s needs’” (Jameton, 1993). Literature since has discussed relations to, and distinctions with, clinician burnout (e.g., Dean et al., 2019; National Academy of Medicine, 2019).

Subsequent authors have extended the concept of moral distress to capture a broad array of events and situations that are “morally undesirable” (Campbell et al., 2018). Rather than focusing on the conditions and nature of the initial moral event, they define distress in terms of the specific psychological responses to the moral events. Moral events broadly include distress as defined by Jameton as well as dilemmas, conflicts, uncertainties, other constraints, and various situations that may threaten to undermine moral integrity (Kälvemark et al., 2004; Fourie, 2015; Campbell et al., 2018; Morley et al., 2021). Such an understanding effectively subsumes, but extends beyond, Jameton’s definition. While the scientific moral distress literature clearly originated in the study of the experience of nurses, the phenomenon itself, under this broad definition, is much more generally applicable. We would thus propose defining moral distress, capturing these more general phenomena, as:

*Moral Distress: Distress that arises because personal experience disrupts or threatens: (a) one’s sense of the goodness of oneself, of others, of institutions, or of what are understood to be higher powers, or (b) one’s beliefs or intuitions about right and wrong, or good and evil.*

The distinction between this more general notion of moral distress, and the definition of moral injury noted earlier is the *persistence* of the distress (i.e., moral injury is *persistent* moral distress). With this broader understanding of moral distress, we see moral distress and moral injury as part of a broader spectrum (cf. Grimell and Nilsson, 2020; Rosen et al., 2022), which we will refer to as “moral trauma,” and we will consider this spectrum in greater detail in the following section.

The definition of moral distress above captures the definition put forward by Jameton wherein the distress arising from institutional constraints hinders one from acting morally, and potentially threatens a person’s sense of the goodness of oneself and/or that of others or of the institution itself. However, our proposed definition of moral distress covers a host of other cases as well as might arise from a moral dilemma, the perpetrating of a transgression, or the witnessing of some traumatic event. Our definition we believe corresponds more closely to the ordinary language understanding of “moral distress” of which the sort of institutional moral distress described by Jameton is an example. Such institutional distress in which the distress arises in response to one’s perceived incapacity to fulfill moral obligations due to institutional, professional, or societal constraints is of course an important and sadly pervasive example of the broader class of moral distress. However, the term “moral distress” as such has wider applicability and conceptual coverage than the institutional moral distress Jameton described.

## 4 The spectrum of moral trauma

### 4.1 Morally traumatic experience and moral injury disorder

In placing moral distress and moral injury on a spectrum of moral trauma, we might also consider the fuller range of this proposed spectrum (cf. Grimell and Nilsson, 2020; Rosen et al., 2022). We will first provide a definition concerning the experiences giving rise to moral distress and will then consider cases in which moral injury is so severe as to involve considerable functional impairment which, under certain circumstances, might be considered a “moral injury disorder.” However, we will comment below on legitimate concerns that may be raised about potentially pathologizing the phenomenon of moral injury, and the additional work that would need to be done if this were to be recognized as a clinical diagnosis, and under what criteria, rather than simply as a descriptive term.

Certain moral events or experiences, as noted above, have the potential to give rise to moral distress and moral injury. They may lead to moral injury in certain cases, but may not do so in others. In the moral injury literature, the moral event itself that may, or may not, potentially give rise to such moral injury and distress is sometimes referred to as a potentially morally injurious event (PMIE). Whether such events give rise to persistent moral distress will depend in part on an individual’s moral understanding, the certainty of moral beliefs, an understanding of their own identity, and a host of other factors. A potentially morally injurious event may give rise to distress for certain people, but may not for others.

The event itself has an objective character and can be described simply in terms of what took place; the experience of that event has a subjective character and it may or may not give rise to moral distress or moral injury. When the experience of the event does give rise to distress by threatening or disrupting one’s moral understanding, it makes sense to refer to this as a “morally traumatic experience” (MTE). The trauma is no longer potential, but actual. Drawing upon our definitions above we would thus propose defining a morally traumatic experience (MTE) as follows:

*Morally Traumatic Experience: Personal experience that disrupts or threatens: (a) one’s sense of the goodness of oneself, of others, of institutions, or of what are understood to be higher powers, or (b) one’s beliefs or intuitions about right and wrong, or good and evil.*

The definition parallels our proposed definitions of moral injury and moral distress except that rather than making reference to *distress*, it instead refers to the *experience* which threatens or disrupts one’s moral understanding. The focus is the experience rather than the distress. However, it is also the case that any threatening or disruption of one’s moral understanding arguably does itself constitute a form of distress. The morally traumatic experience is the origin of the ensuing moral distress, which, if persistent, constitutes moral injury.

In certain cases, a morally traumatic experience may cause moral distress sufficiently persistent and severe that it may substantially impede one’s capacity to function. If such impairments are severe and understood as being well outside the range of societal norms, it might then be reasonable to speak of a moral injury *disorder*. Consequently, we propose defining a moral injury disorder as:

*Moral Injury Disorder: Persistent distress that arises because personal experience disrupts or threatens: (a) one’s sense of the goodness of oneself, of others, of institutions, or of what are understood to be higher powers, or (b) one’s beliefs or intuitions about right and wrong, or good and evil, so as to cause impairment in social, occupational, or other important areas of functioning in ways that are out of proportion or inconsistent with cultural or religious norms concerning such experiences.*

The restriction here that the impairment in functioning is “out of proportion or inconsistent with cultural or religious norms concerning such experiences” rules out certain cases in which cultural norms are such as to involve expectations of voluntary withdrawal from certain forms of activity or normal function. The restriction here is analogous to similar criteria in Prolonged Grief Disorder diagnosis of DSM-5, so as to exclude relatively normal cases of bereavement. As discussed further below, considerable additional research would be required to determine whether this category of moral injury disorder in fact has clinical utility, and whether it would be reasonable for this to be recognized as a diagnostic category, and under what criteria, rather than simply a descriptive term.

### 4.2 Persistence and severity

**Figure 1:**
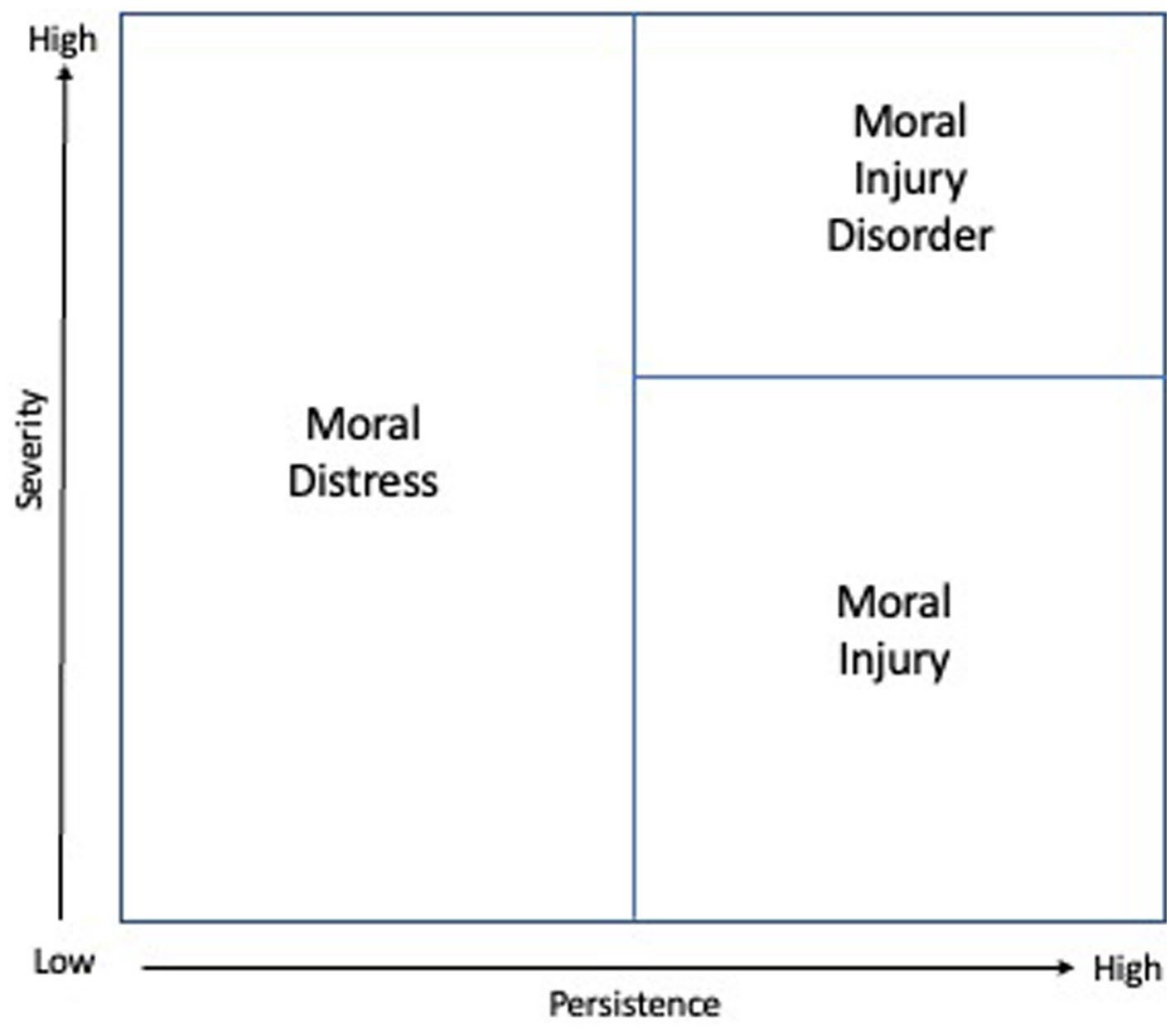
The moral trauma spectrum in terms of persistence and severity.

In considering the relation between our various definitions above, it should be noted that there are effectively two dimensions being employed in what we are conceiving of as a “moral trauma spectrum”: persistence and severity. It is persistence that distinguishes “moral injury” from the less persistent forms of “moral distress.” It is severity of impairment that distinguishes “moral injury disorder” from less severe forms of “moral injury.” Figure 1 plots the extent of severity from low to high, and persistence from low to high. Instances with relatively low persistence would not constitute moral injury, but moral distress. Instances with high persistence can constitute moral injury, and when the severity of that injury is substantial, it might be reasonable to even speak of “moral injury disorder” but we will return to the complexities of such a designation below. It is, however, also possible in principle to experience severe forms of moral distress that are not persistent, and we will also consider such cases further below.

With the understanding presented, the term “moral trauma” may thus refer either to the *experience* that gives rise to moral distress, *or* to any of the more or less persistent or severe, forms of the *moral distress* itself. The experience and the distress are conceptually distinct categories, and the moral trauma spectrum concerns the persistence and severity of the distress itself. However, both the experience and the distress would arguably be appropriately referred to as “moral trauma” in ordinary language. The definition of “trauma” in the Oxford English Dictionary includes both an “external bodily injury” and “the condition caused by this;” in the context of “moral trauma” the analogues are thus arguably either the disruptive moral experience itself, or the condition of moral distress caused by it.

Given the definitions described thus far, one might in principle make reference to “mild moral distress,” if the ensuing distress is not particularly severe, and one might then question whether it is appropriate to refer to such mild distress as “trauma.” However, we would argue that the very nature of personal experience that “disrupts or threatens… one’s sense of the goodness of oneself, of others, of institutions, or of what are understood to be higher powers… or one’s beliefs or intuitions about right and wrong, or good and evil” is itself sufficiently severe so as to warrant that the experience itself, or any moral distress ensuing from it, being referred to as “trauma.” However, others might reasonably prefer to refer to such instances as “moral pain” or “moral stress.”

While Figure 1 represents severity as a single dimension, this severity might itself be considered separately in at least three distinct aspects: (i) the severity of the disruption to one’s moral understanding, (ii) the severity of the distress or symptoms, and (iii) the severity of the impairment. These three aspects of severity are of course interrelated. The more severe the disruption to one’s moral understanding, the greater we might expect the distress and symptoms to be; and the more severe the distress and symptoms, the greater we might expect the functional impairment to be. However, other aspects of a person’s temperament, understanding, resources, and social community might alter the relationship among these various aspects of severity, and so the three aspects might in principle be considered separately. It is also moreover the case that each of these three aspects of severity is itself multidimensional: the disruption may concern one’s sense of the goodness of oneself, of others, of institutions, or of God or one’s beliefs or intuitions about right and wrong, or good and evil. The distress may involve guilt, shame, betrayal, anger, or other moral emotions. The impairment may concern one’s work, or social relationships, or other areas of day-to-day functioning. These three aspects of severity –extent of moral disruption, distress symptoms, and functional impairment– along with the multidimensional nature of each aspect, will be important in considering assessments for the various forms of moral trauma below.

Moral injury disorder, as the extreme pole of moral trauma and as involving considerable functional impairment, would arguably constitute grounds for clinical care or counseling. However, even moral distress or injury which does not constitute “disorder,” might also often warrant clinical care or counseling, a point to which we return below. Furthermore, as indicated in the Figure, moral distress itself, even if it is not persistent, can nevertheless potentially be severe. Such severe forms of moral distress may likewise also sometimes merit clinical care or counseling, even if not persistent and not constituting (or not yet constituting) a “moral injury.” Clinical attention may be warranted for temporary but severe forms of moral distress, both because of the potential effects of the severity of the distress on a person’s life, and also because if such severe distress is not addressed, it may well continue to persist and thus subsequently develop into what would constitute a moral injury.

## 5 Distinctions with PTSD

Moral injury is often studied within the context of events and experiences that might also give rise to post-traumatic stress disorder (PTSD). The degree of overlap and distinctions with PTSD should thus also be considered. While further empirical study is needed, arguments and evidence have begun to accumulate that the phenomenon can in fact be distinguished from PTSD (Litz et al., 2009; Bryan et al., 2018; Litz and Kerig, 2019; Griffin et al., 2019; Koenig et al., 2019). First, there are important conceptual distinctions. For example, PTSD, at least in its current clinical understanding (both DSM-V and also each of PTSD and Complex PTSD in the ICD-11), must involve a persistent re-experiencing of the traumatic event, whereas moral injury may or may not. Likewise, PTSD requires avoidance of trauma-related stimuli following the trauma, whereas once again moral injury may or may not. Relatedly, a major component of PTSD concerns fear, which may or may not play a substantial role in moral injury and moral distress (Litz et al., 2009).

Conversely, moral injury and moral distress must always concern some action or experience related to moral valuation or worth, whereas PTSD may or may not. The witnessing of a natural disaster, for example, might for many give rise to PTSD; the event itself might be interpreted by some in a manner wherein there are implications for one’s entire moral framework, but it may not be thus interpreted by others. When the witnessing of such an event is not interpreted in moral terms, it might nevertheless still result in PTSD because of a continually revisiting of the trauma, but it would not, in these cases, constitute moral injury.

Further evidence for a distinction arises from differences in what at present are considered preferred approaches to treatment for PTSD and moral injury. For example, prolonged exposure therapy -- a common treatment for PTSD – will not be especially helpful for treating moral injury if questions of guilt or shame or betrayal are not addressed (Griffin et al., 2019). Alternative treatments such as adaptive disclosure therapy (Litz et al., 2016) or approaches that incorporate aspects of spirituality or forgiveness (Koenig and Al Zaben, 2021) that more directly address moral concerns may be more effective in addressing moral injury than, for instance, in cases of PTSD in which devastating events are less connected with moral transgressions.

Even biologically, the phenomena seem to pertain to different parts of the brain. Whereas PTSD involves impaired prefrontal cortex and an overactive amygdala, resulting in fight or flight reactions in non-dangerous “trigger situations,” with moral injury the prefrontal cortex (where moral thinking occurs) must be intact. For example, damage to the right frontotemporal cortex, as occurs in frontotemporal dementia, is associated with a loss of moral behavior (Roberts et al., 2019) and the person who does not experience emotional consequences of transgressing moral values has diminished functioning of the frontal cortex based on MRI studies of their brain. These clinical and neuroanatomical findings again suggest that PTSD and moral injury are separate but sometimes overlapping conditions.

Of course, there is certainly some overlap in symptoms between moral injury and PTSD. However, this overlap concerns principally the PTSD Criterion D symptoms concerning negative alterations in cognitions and mood (Koenig et al., 2019). There would be similar overlap with symptoms of major depression as well. Overlap of symptoms between different mental disorders is not unusual. These overlapping symptoms, however, do not define moral injury, which is instead characterized by a unique set of experiences concerning threat or disruption of one’s moral system, concerning which there is no necessary overlap with symptoms of depression or anxiety or PTSD.

However, although these distinctions can be drawn, there will nevertheless often be cases in which the same event gives rise to both PTSD and moral injury. In certain cases, an event may prompt either PTSD or moral injury which might then in turn also lead to the other. For example, an event itself may not initially threaten a person’s moral understanding, but perhaps by the repeated re-experiencing of the trauma over time, a person’s sense of right or wrong is eventually threatened or disrupted, then further resulting in moral injury. Conversely, it is possible that a particular transgression immediately triggers deep moral concerns and the persistent distress over these concerns does eventually, but not immediately, result in a persistent fear over, and re-experiencing of, the trauma itself. Thus, while PTSD and moral injury are arguably distinct, the same event might simultaneously give rise to both, or alternatively, one phenomenon might give rise to the other. The work of Barr et al. (2022) and Atuel et al. (2021) concerning a “dual process model” provides a potentially promising approach for understanding the relations between the two.

In proposing the more extreme category of the moral trauma spectrum of “moral injury disorder,” there is, of course, some risk in pathologizing the very notion of moral injury (Farnsworth et al., 2017, 2019). Such pathologizing might shift the focus to more clinical forms of care on matters that are perhaps better addressed through pastoral care or counseling, or it might risk eventually leading to reliance on pharmacological treatments in place of counseling, or might render more likely the ignoring of the context of the injury, or might lead to greater stigma (Kinghorn, 2020). There is moreover ambiguity as to what is “out of proportion or inconsistent with cultural or religious norms,” and it is not clear that determinations about such ambiguity will necessarily be appropriately made in clinical settings. However, the literature on moral injury now seems sufficiently advanced so as to make clear that there are at least some instances in which the moral struggles a person faces pose persistent and considerable challenges to their functioning, challenges that are distinct from, and often are not addressed by, treatment for PTSD. It seems important then to acknowledge the persistence and severity of impairment that such moral struggles can sometimes give rise to. The resolution of some of the concerns above is arguably not to abandon moral injury as a condition requiring care and treatment, but rather to include within such treatment pastoral care and counseling that addresses the specifically *moral* nature of the distress. Ideally, a team of chaplains, behavioral health specialists, and physicians would together provide such care. An acknowledgement that sometimes moral injury can be sufficiently severe so as to lead to such impairment would arguably facilitate attention to, and care for, those experiencing such severe moral distress.

## 6 Proposed moral trauma assessment

### 6.1 Proposed moral trauma assessment items

In what follows, based on our discussion above, we will propose a series of items that can be used as a tool for assessing the spectrum of moral trauma. Following Carey and Hodgson (2018) and the discussion of our definitions above, we divide the principal assessment items into the definitional aspects (which characterize moral injury itself) and the accompanying symptoms. Contrary to prior assessments, we will focus our symptom assessment on those that Carey and Hodgson (2018) refer to as “core symptoms” (i.e., those that are constitutively connected with the moral disruption or threat). These include feelings of guilt, shame, betrayal, loss of trust, etc. What Carey and Hodgson (2018) refer to as secondary symptoms may, and often will, arise from moral trauma but are arguably the effects of moral trauma, but not constitutive aspects. However, it would often be appropriate to also include other survey assessments for these secondary symptoms, for example for depression, anxiety, and PTSD, that may result from moral trauma, using for instance, the PHQ-9 (Kroenke et al., 2001) or the PCL-5 (Blevins et al., 2015), or others.

We put forward seven definitional moral trauma items and ten symptom items. These items were proposed and refined during regular meetings of the authorship team over the course of more than a year, based on the items’ content validity and prior item proposals, with subsequent refinement in light of the proposed definitions above, and with a focus also on symptom items that best represented the emotions that individuals with a healthy conscience experience when they transgress, observe others transgress, or experience transgression of their moral values. Further cognitive testing and psychometric work will evaluate the assessment’s diagnostic and research utility.

The first six of the seven definitional moral trauma items are derived from the personal experience being one that “disrupts or threatens one’s sense of the goodness of oneself, of others, of institutions, or of what are understood as higher powers,” or “one’s beliefs or intuitions about right and wrong, or good and evil.” The first item concerns good and evil, the second concerns right or wrong, the third through sixth relate, respectively, to the goodness of oneself, of others, of institutions, or of the divine. If the assessment is used purely for clinical purposes rather than for research, items four through six might be combined into, “Because of what I experienced, I question the goodness of others, of institutions, or of the divine.” The final seventh item is intended to capture a sense of persistent distress. Without an endorsement of at least one of items one through six, the experience would arguably not be one of moral trauma, and without also an endorsement of item seven, the phenomenon would arguably not be one of *ongoing* moral distress or injury.

With respect to the symptoms, none of these individually are necessary, though the absence of all of them would generally indicate that distress arising from the moral nature of the experience was minimal. These ten core symptom items concern guilt, shame, betrayal, anger, powerlessness, hopelessness, loss of meaning, struggles with faith, struggles with forgiveness, and loss of trust. Guilt, shame, betrayal, and anger are often included as symptoms constitutively related to moral injury or moral distress. However, the disruption or threatening of one’s moral framework might well also constitutively relate to powerlessness, hopelessness, loss of meaning, struggles with faith, struggles with forgiveness, and loss of trust in that these may be entailed by the particular aspect of a person’s moral framework that has been threatened or disrupted. Note that our proposed definition of moral injury and our assessment allows for more “religious” forms of moral injury that might result in struggles with faith, but it in no way requires that these be present.

We first consider these symptom items for moral trauma generally, regardless of whether the experience concerns that of a perpetrator, witness, victim, or something other than an interpersonal transgression. We will later consider modifications of these items that are more specifically tailored to contexts of perpetrator, witness, or victim.

Before employing the assessment, we would propose asking a screening question to determine whether there is a potential event in view that may have given rise to moral distress. Examples of such a screening question might be “Is something severely disrupting your moral beliefs or your sense of goodness of yourself or others?” or alternatively “Have you done or experienced something that is severely disrupting your moral beliefs or your sense of goodness of yourself or others?” If the answer to such a question is “yes,” then it may be reasonable to proceed with the moral trauma assessment. When appropriate, such a screening question could be followed up with a further question, “Would you like to describe what took place, or what you did, or what the experience was?” so as to better understand the nature of the morally traumatic experience.

For the seven definitional moral trauma items we would propose response categories of: “disagree, somewhat agree, agree, strongly agree.” For the ten symptom items we would propose responses on a 0–10 scale ranging from 0 = “Strong Disagree” to 10 = “Strongly Agree.” In subsequent work we will subject the items and response categories to cognitive testing and to psychometric evaluation and will consider alternative response categories. The proposed seven definitional items and the ten moral trauma symptom items are listed in Table 1.

**Table 1:** Proposed moral trauma definitional and symptom items^†^.

Moral trauma definitional items (regardless of moral trauma type):
Because of what I experienced, I question my beliefs about good and evil.
Because of what I experienced, I feel more confused about right and wrong.
Because of what I experienced, I doubt my own personal goodness.
Because of what I experienced, I question the goodness of others.
Because of what I experienced, I question the goodness of institutions.
Because of what I experienced, I question the goodness of God or of higher powers.
I am still troubled over what took place.
Moral trauma symptoms (regardless of moral trauma type):
I feel guilt over what occurred.
I feel ashamed over what happened.
I feel that I have been betrayed or that I have betrayed myself.
I am angry over the wrong that occurred.
I feel powerless to act rightly after having experienced what I did.
I feel a sense of hopelessness over what happened.
I feel a loss of meaning or purpose because of what took place.
I am struggling with my faith because of what occurred.
I am struggling to forgive others or myself because of what took place.
I am struggling to trust others or myself because of what took place.

^†The seven definitional items have responses of: “disagree, somewhat agree, agree, strongly agree.” The ten symptom items have 0–10 responses ranging from 0 = “Strong Disagree” to 10 = “Strongly Agree”.^

If it is clear that the experience in view is that of a perpetrator, a witness, or a victim, and clear also that the experience is not a combination of these, then the ten symptom assessment items above could be replaced by items specifically tailored to each of the perpetrator, witness, or victim categories. We present our proposal for each of these three ten-item sets in Table 2.

**Table 2:** Moral trauma perpetrator, witness and victim symptoms^†^.

Moral trauma perpetrator symptoms:
I feel guilt over having acted immorally.
I feel ashamed about what I did or did not do.
Because I acted wrongly, I have betrayed who I am as a person.
I am angry at myself for having acted wrongly.
I now feel powerless to act rightly because of what I did.
I am filled with hopelessness because of my wrong action.
Because of my actions I have lost my sense of meaning or purpose.
I am struggling with my faith because I did what was wrong.
I am struggling to forgive myself because of what I did.
Because of how I acted, I feel I can no longer trust myself.
Moral trauma witness symptoms:
I feel guilt about not stopping the wrong that occurred.
I feel ashamed at having witnessed what I saw.
I feel betrayed by those I once trusted.
I am angry over having encountered the wrong that took place.
Because of the wrong that occurred, I now feel powerless to act rightly.
I experience a sense of hopelessness when I think about what I’ve seen.
Because of what took place, I feel a loss of meaning or purpose.
I am struggling with my faith because of what I have witnessed.
I am struggling to forgive those who have done wrong.
Because of what I’ve seen, I feel I can no longer trust others or myself.
Moral trauma victim symptoms:
I feel what happened to me was somehow my own fault.
I fear that others will think differently of me because of the wrong(s) that took place.
I feel betrayed by those I once trusted.
I am angry over the wrong that was done to me.
Having been wronged, I now feel powerless to act rightly.
I experience a sense of hopelessness when I think about what happened to me.
I feel a loss of meaning or purpose because of what was done to me.
I am struggling with my faith because of the wrong that was done to me.
I am struggling to forgive the person who wronged me.
Because of the wrong(s) that occurred, I feel I can no longer trust myself, or others.

^†If it is clear that the experience in view is that of a perpetrator, a witness, or a victim, and clear also that the experience is not a combination of these, then the ten symptom assessment items in Table 1 could be replaced by items specifically tailored to each of the perpetrator, witness, or victim categories in Table 2.^

The items in Table 1 would constitute a general moral trauma assessment. As noted above, endorsement of at least one of the first six definitional items with at least “somewhat agree” would constitute some form of moral trauma. Responses of “agree” or “strongly agree” would indicate a greater severity in terms of the extent of the disruption. Provided the event or experience in view was sufficiently far in the past, an endorsement also of the seventh definitional item would then also constitute moral injury. Responses to the ten symptom items would allow for a fuller assessment of the nature and severity of the distress in terms of its symptoms.

### 6.2 Persistence and impairment

Upon completion of the symptom items a further question could be asked concerning persistence such as, “Of the problems that you indicated were present, how long have you been experiencing these?” Responses to this question could either be open-ended or chosen from response categories of “A few days, a few weeks, 1–2 months, 3–5 months, 6–12 months, or more than a year.” Such supplemental questions would provide greater detail on the persistence of the moral trauma. In research contexts, separate repeated questions about persistence could be asked for each of the symptoms that was being experienced. In cases in which agreement with the core definitional items or symptoms items quickly dissipated over time, it would arguably be more appropriate to refer to the experience as moral distress rather than moral injury.

An additional question could also be asked concerning impairment. Following the analogous wording of PHQ-9 (Kroenke et al., 2001), impairment could be assessed with the question, “How difficult have these problems made it for you to do your work, take care of things at home, or get along with other people?” with response options, “Not difficult at all, somewhat difficult, very difficult, extremely difficult.” In research contexts, the impairment question might be asked separately for each symptom item that was endorsed. We noted above that severity itself can be considered along the lines of several dimensions including (i) the severity of the disruption to one’s moral understanding, (ii) the severity of the distress or symptoms, and (iii) the severity of the impairment. The first of these can potentially be operationalized with the definitional items in Table 1, the second with the symptom items in Table 1, and the third with the impairment questions just mentioned. However, even the response to this impairment question would not definitively allow for assessing moral injury disorder, as defined above, as it does not allow for determining whether the impairment is “out of proportion or inconsistent with cultural or religious norms.” However, it would potentially allow for assessing whether further inquiry or clinical care might be warranted.

Our proposed moral trauma assessment is also arguably applicable in the context of moral distress from institutional constraints imposed upon nurses (Jameton, 1984) insofar as an endorsement of the fourth definitional core item, “Because of what took place, I question the goodness of institutions,” would be appropriate, potentially followed by assessment of the various symptoms. However, the use of the items above for assessing the narrower notion of moral distress, as conceived by Jameton, would constitute a departure from the current moral distress assessment literature which is focused only on moral distress arising from institutional constraints. Jameton’s definition guided the development of measures for moral distress, which focused heavily on the nature of the constraints that cause moral distress (Corley et al., 2001; Deschenes et al., 2020; Morley et al., 2021). Constraints were conceptualized as both institutional and individual factors that create the ethically challenging conditions of moral events (Wilkinson, 1987). In the Measure of Moral Distress – Healthcare Professionals (MMD-HP), for example, individuals rate the frequency with which constraining events occur and the subjective severity of distress the situation caused them (Epstein et al., 2019). If assessment or research were being carried out in a nursing setting that was clearly focused upon institutional constraints, then it would often be preferable to use prior assessments developed specifically for this purpose. Our proposed assessment is intended to cover a much broader range of phenomena that might be classified as moral distress or moral trauma. Nevertheless, even in a nursing setting, if forms of moral distress were of interest beyond those arising from institutional constraints, as might occur for example, in cases of a genuine moral dilemma, a legitimate medical error, or the unexpected death of a patient, then our more general assessment might still be of interest.

### 6.3 Future work

Further psychometric work will be needed to evaluate the properties of the proposed assessment. Further research would also be required for determining whether reasonable empirically grounded distinctions between moral distress, moral injury, and moral injury disorder can be drawn from the proposed assessment. The assessment would provide a place to begin to explore issues of validation of these categories and this may in turn offer opportunities for the refinement of the assessment and we intend to carry out this work in the years ahead. Further work would also be needed to establish whether the category of moral injury disorder is itself of clinical utility, and whether it would be reasonable for this to be recognized as a diagnostic category, and under what criteria, rather than simply a descriptive term.

As a closely relevant development, in December 2024, the American Psychiatric Association (APA) took an historic step by approving the addition of “Moral” to the existing “Religious or Spiritual Problem” category in the Section of Other Conditions That May Be a Focus of Clinical Attention in the DSM-5-TR. This action, approved at the APA Board of Trustees’ December 13, 2024 meeting, is a crucial step toward recognizing and treating moral injury and moral distress. The language of the DSM-5-TR now reads:

Z65.8 Moral, Religious, or Spiritual Problem This category may be used when the focus of clinical attention is a moral, religious, or spiritual problem. Moral problems include experiences that disrupt one’s understanding of right and wrong, or sense of goodness of oneself, others or institutions. Religious or spiritual problems include distressing experiences that involve loss or questioning of faith, problems associated with conversion to a new faith, or questioning of spiritual values that may not necessarily be related to an organized church or religious institution.

The DSM-5-TR language for “moral problem” corresponds closely to what we have proposed above as a “morally traumatic experience,” and instances of what we have defined above as “moral injury” would always constitute under the DSM-5-TR language a “moral problem.” According to the DSM-5-TR, Z-codes, such as that above, are “conditions and psychosocial or environmental problems that may be a focus of clinical attention or otherwise affect the diagnosis, course, prognosis, or treatment of an individual’s mental disorder… The conditions and problems… are not mental disorders. Their inclusion in DSM-5-TR is meant to draw attention to the scope of additional issues that may be encountered in routine clinical practice and to provide a systematic listing that may be useful to clinicians in documenting these issues.” Nevertheless, this DSM-5-TR Z code does open important opportunities and pathways for awareness and treatment of moral trauma, moral distress, and moral injury.

## 7 Conclusion

In this paper we have put forward the notion of “moral trauma” as a spectrum to cover moral injury and moral distress, broadly conceived, and that might be extended to moral injury disorder. Our definition of moral injury expands prior consensus-based definitions to more fully include moral injury arising from being a victim. Our moral trauma assessment can be used to assess moral injury and is applicable to perpetrators, witnesses, victims, or more generally, and might also be used to assess less persistent or less severe forms of moral distress. Further cognitive testing and psychometric work will be needed to evaluate its diagnostic and research utility, as well as its potential to assess cases of moral injury or moral injury disorder of sufficient severity so as to merit clinical care or clinical pastoral counseling.

## Footnotes

^1^One possible exception might appear to be experiences wherein there are “moral transgressions… which fundamentally challenges the moral integrity of an… organization or community” (Carey and Hodgson, 2018) but seemingly do not challenge the moral integrity of the individual. However, we believe that this too would be covered by our definition insofar as it would thereby threaten or disrupt the individual’s sense of the goodness of that institution or community.
